# MKado: a toolkit for McDonald–Kreitman tests of natural selection

**DOI:** 10.1093/g3journal/jkag142

**Published:** 2026-06-01

**Authors:** Angel G Rivera-Colón, Clara T Rehmann, Andrew D Kern

**Affiliations:** Institute of Ecology and Evolution and Department of Biology, University of Oregon, Eugene, OR 97403, United States; Institute of Ecology and Evolution and Department of Biology, University of Oregon, Eugene, OR 97403, United States; Institute of Ecology and Evolution and Department of Biology, University of Oregon, Eugene, OR 97403, United States

## Abstract

MKado is a Python toolkit for performing McDonald–Kreitman (MK) tests of natural selection from aligned coding sequences. It implements the standard MK test as well as a wide variety of its extensions and related statistics, including a number of methods for estimating the fraction of adaptive substitutions (*α*) while accounting for slightly deleterious mutations, with a unified command-line interface and Python API. MKado supports parallel batch processing of thousands of genes with near-linear scaling, and provides publication-ready visualizations including volcano plots and asymptotic *α* curves.

## Teaser

The McDonald-Kreitman test is a widely-used statistical method that helps scientists detect natural selection in DNA sequences by comparing genetic differences within and between species. However, existing software tools for this test are scattered across different programs and lack modern features. MKado is a new, freely available Python toolkit that brings together the McDonald-Kreitman test and its many extensions into one easy-to-use package. It can analyze thousands of genes efficiently, includes advanced statistical methods for measuring adaptation, and creates publication-ready figures. This tool will help evolutionary biologists more easily study how natural selection shapes genetic variation across diverse organisms.

## Introduction

The McDonald–Kreitman (MK) test (McDonald and Kreitman 1991) is among the most widely used methods for detecting natural selection at the molecular level. By comparing the ratio of nonsynonymous to synonymous changes within species (polymorphism) to that between species (divergence), the MK test provides a direct test of the neutral theory prediction that these ratios should be equal. Departures from this expectation, often quantified by the neutrality index (NI; Rand and Kann 1996) or the fraction of adaptive substitutions *α* (Smith and Eyre-Walker 2002), have revealed pervasive positive selection across a wide range of taxa (e.g. Hughes et al. 2008; Li et al. 2008; Downing et al. 2009; Liti et al. 2009; Lam et al. 2010; Lozada-Chávez et al. 2025; Brown et al. 2024).

A key challenge for the standard MK test is that slightly deleterious nonsynonymous mutations, which contribute to polymorphism but rarely fix, inflate the number of nonsynonymous polymorphisms relative to nonsynonymous substitutions and bias *α* downward. Early approaches addressed this by excluding low-frequency polymorphisms, either by distinguishing tip from interior mutations on the gene tree (Templeton 1996) or by applying a derived allele frequency cutoff (Fay et al. 2001). The asymptotic MK test further addresses this limitation by estimating *α* as a function of derived allele frequency and extrapolating to fixation, effectively removing the contribution of weakly deleterious variants (Messer and Petrov 2013; Haller and Messer 2017). Rather than discarding low-frequency polymorphisms entirely, the imputed MK test uses the ratio of low- to high-frequency synonymous polymorphisms to estimate the expected number of weakly deleterious nonsynonymous polymorphisms and subtracts them from the total, retaining more data while correcting for the bias (Murga-Moreno et al. 2022). To address lineage-specific selection, the polarized MK test uses a second outgroup to assign fixed differences to specific lineages, allowing lineage-specific inference of selection (Begun et al. 2007).

Despite the centrality of MK-based methods to molecular evolution, existing software implementations remain fragmented. DnaSP (Librado and Rozas 2009) provides the standard test through a Windows-only graphical interface. The iMKT R package and web server (Murga-Moreno et al. 2019) implements several extensions but requires pre-computed summary statistics and is tightly coupled to specific databases. The asymptoticMK web tool (Haller and Messer 2017) implements the asymptotic test but does not accept raw sequence data. The degenotate software, part of the snpArcher pipeline Mirchandani et al. (2023), can calculate both standard and imputed versions of the test; however it does so only using genetic variants in VCF format and is thus dependent on (and susceptible to) the liftover of outgroup sequences. No existing tool provides a unified command-line interface that takes aligned coding sequences as input and supports the standard, polarized, and asymptotic MK tests together with batch processing across thousands of genes (Table 1).

**Table 1.** Feature comparison of MK test software. Checkmarks indicate built-in support; dashes indicate the feature is absent.

Feature	DnaSP	iMKT	asymptotic MK	degenotate	MKado
Sequence (FASTA) input	✓	web only	—	—	✓
VCF input	—	—	—	✓	✓
Standard MK	✓	✓	—	✓	✓
Polarized MK	—	—	—	—	✓
Asymptotic MK	—	✓	✓	—	✓
Imputed MK	—	✓	—	✓	✓
$\alpha_{\mathrm{TG}}$	—	—	—	—	✓
Frequency filtering	—	✓	✓	✓	✓
CLI	—	—	—	✓	✓
Python API	—	—	—	—	✓
Batch processing	—	—	—	—	✓
Parallel execution	—	—	—	—	✓
Multiple testing corr.	—	—	—	—	✓
Visualization	limited	✓	✓	—	✓
Open source	—	✓	✓	✓	✓
Cross-platform	—	✓	✓	✓	✓

Here we present MKado, a Python package that fills this gap. MKado takes both codon-aligned FASTA and VCF files as input and implements the standard, polarized, asymptotic, and imputed MK tests, as well as related statistics, with a consistent command-line interface. It supports parallel batch processing of genome-scale datasets, automatic multiple testing correction, and built-in visualization including volcano plots and asymptotic *α* curves. MKado is installable via pip and is designed to integrate naturally with modern Python-based bioinformatics workflows.

## Features and implementation

MKado is implemented in Python and provides both a command-line interface built on Typer and a Python API for programmatic use. It accepts codon-aligned FASTA files as input, requiring no pre-processing or external format conversion. Sequences are automatically partitioned into ingroup and outgroup sets by name pattern matching, supporting both multi-species alignments in a single FASTA file and separate files containing ingroup or outgroup sequences. MKado also accepts variant-call input via the vcf subcommand, which uses a multi-sample ingroup VCF, a single-sample outgroup VCF, a reference FASTA, and a GFF3 annotation to derive codon-level polymorphism and divergence counts directly and supports the full set of MK analyses available to the FASTA workflow. Together, these two input modes distinguish MKado from FASTA-only tools (DnaSP, asymptoticMK) and from VCF-only tools (degenotate, fastDFE). Full documentation, including tutorials and API reference, is available at https://mkado.readthedocs.io.

### Analysis methods

MKado implements five MK-based analyses. The *standard* test computes the classical 2×2 table of nonsynonymous and synonymous changes in polymorphism and divergence, with significance assessed by Fisher’s exact test or the *G*-test. $D_{n}$ and $D_{s}$ are the numbers of fixed differences between ingroup and outgroup at codon positions where the inferred substitution is nonsynonymous or synonymous, respectively, under the supplied genetic code. $P_{n}$ and $P_{s}$ are the corresponding numbers of ingroup polymorphic sites at which the derived (non-outgroup) allele segregates above an optional frequency threshold (default zero, i.e. all polymorphisms; --min-freq *X* excludes sites with derived allele frequency below *X*, and --no-singletons excludes singletons by setting the threshold to 1/n for sample size *n*). By default $P_{n}$ and $P_{s}$ follow the DnaSP convention and exclude sites that also segregate in the outgroup; --pool-polymorphisms pools ingroup and outgroup polymorphism instead. Summary statistics include the neutrality index (NI; Rand and Kann 1996), *α* (Smith and Eyre-Walker 2002), and the Direction of Selection statistic (DoS; Stoletzki and Eyre-Walker 2011). Tests that depend on derived allele frequency—the asymptotic and imputed MK tests described below—require knowing the derived allele at each polymorphic site. MKado polarizes alleles automatically using the outgroup: at each polymorphic site, the allele matching the outgroup is treated as ancestral and any non-outgroup allele as derived. This automatic single-outgroup polarization is distinct from the *polarized* MK test below, which uses a *second* outgroup to attribute each fixed difference to either the ingroup or the outgroup lineage. The *polarized* test uses a second outgroup to infer the ancestral state at each fixed difference, assigning divergence to the ingroup or outgroup lineage for lineage-specific inference. The *asymptotic* test bins polymorphisms by derived allele frequency, computes *α* within each bin, and fits an exponential model $\alpha (x)=a+be^{-cx}$ to extrapolate the asymptotic *α* at x=1, correcting for the contribution of weakly deleterious variants (Messer and Petrov 2013). A linear model is also fitted and AIC selects the better specification, following Haller and Messer (2017). MKado supports both the original per-bin SFS construction of Messer and Petrov (2013), in which $P_{n}(x)$ and $P_{s}(x)$ count polymorphic sites with derived allele frequency *equal to x*, and the cumulative inclusive variant of Uricchio et al. (2019), in which $P_{n}(x)$ and $P_{s}(x)$ count polymorphic sites with derived allele frequency *at or above x* (selectable with --sfs-mode {at,above}); the cumulative form has the same asymptote at x=1 but is more robust to bin-count sparsity in large samples. The asymptotic test supports both per-gene and aggregated (pooled across genes) modes; for the aggregated mode, two confidence-interval methods are available (--ci-method): a parametric Monte-Carlo procedure that samples the curve-fit covariance matrix (default; fast) and a case-resampling bootstrap that resamples the pooled polymorphism list and refits per replicate (slower per replicate but more principled when per-bin counts are small). The *imputed* test corrects for weakly deleterious mutations by estimating their contribution from the ratio of low- to high-frequency synonymous polymorphisms (Murga-Moreno et al. 2022). Polymorphisms are split at a derived allele frequency cutoff (default 15%), and the expected number of weakly deleterious nonsynonymous polymorphisms below the cutoff is imputed as $P_{wd}=P_{n,\text{low}}-P_{n,\text{high}}\times (P_{s,\text{low}}/P_{s,\text{high}})$. The corrected count $P_{n}^{*}=P_{n}-P_{wd}$ is then used in the standard 2×2 test to obtain a bias-corrected *α*. This approach retains more data than simple frequency filtering while still removing the downward bias from slightly deleterious variants. Finally, the Tarone-Greenland $\alpha_{\mathrm{TG}}$ estimator (Stoletzki and Eyre-Walker 2011) provides an unbiased multi-gene estimate of *α* by weighting each gene inversely by $P_{s}+D_{s}$, correcting for sample size heterogeneity across loci.

### Codon change classification

When comparing codons that differ at multiple positions, the assignment of changes as synonymous or nonsynonymous is not straightforward because the order of substitutions is unknown. MKado pre-computes all shortest mutational paths between every pair of codons and weights the synonymous and nonsynonymous contributions equally across paths, following the approach of Nei and Gojobori (1986).

### Batch processing and output

MKado’s batch command processes directories of alignment files in parallel using Python’s multiprocessing facilities with configurable worker counts. Benjamini-Hochberg false discovery rate (FDR) *q*-values are computed automatically across all genes. Results are available in human-readable, TSV, or JSON formats. A companion info command reports alignment diagnostics (sequence count, length, and codon structure) for input validation.

To evaluate scaling performance, we benchmarked MKado on 12,437 human protein-coding genes aligned with chimpanzee and orangutan orthologs from OrthoMaM (Allio et al. 2024), with polymorphism data from 178 Yoruba individuals in the 1,000 Genomes Project (1000 Genomes Project Consortium 2015). Processing all genes with 128 worker threads completed in 28 seconds, a ∼50× speedup over single-threaded execution. Per-gene work (alignment parsing, codon classification, and the per-gene MK computation) takes only milliseconds, so the per-process spawn overhead from Python’s multiprocessing grows non-negligible relative to per-task work as the worker count rises. Supplementary Figure S1 shows approximately linear scaling up to roughly 16 workers, with progressively diminishing efficiency past 32 workers; we therefore recommend setting the worker count well below the gene count for batch runs at any scale. For workloads dominated by aggregated tests (asymptotic MK with case-resampling bootstrap CIs), where per-task work is much larger, scaling extends usefully to higher worker counts (Supplementary Fig. S3).

### Visualization

**Fig. 1.**
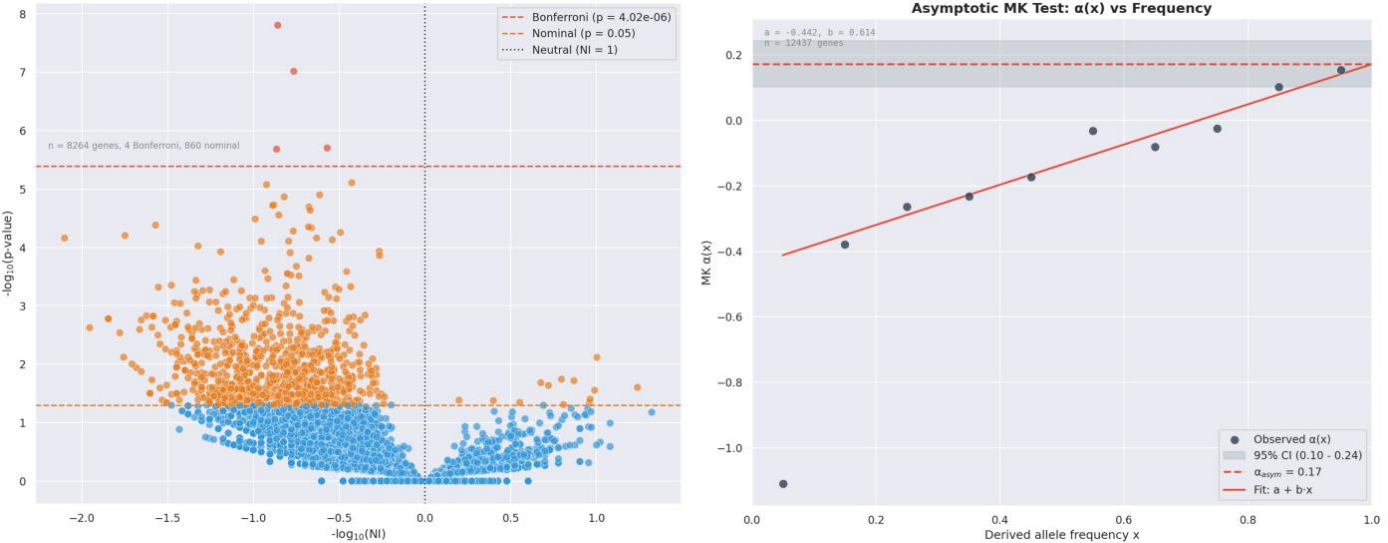
MK test results for 12,437 human protein-coding genes using *Pongo abelii* as the outgroup for divergence. Left: Volcano plot from per-gene standard MK tests. Red points are significant after Bonferroni correction, while orange points are nominally significant (p<0.05). The dashed lines indicate Bonferroni and nominal thresholds. The leftward shift of the cloud (NI >1) reflects the well-known excess of weakly deleterious nonsynonymous polymorphism in humans. Right: Asymptotic *α* curve for pooled data. Points represent the observed *α* at each derived allele frequency cutoff; the solid line represents the fitted curve, in this case the linear model selected by AIC. The shaded area indicates the 95% confidence interval around the fitted asymptotic *α* value.

MKado generates publication-ready plots directly from the command line. Volcano plots display $-\log_{10}\;(\text{NI})$ against $-\log_{10}\;(p)$ for each gene, with Bonferroni and nominal significance thresholds (left panel of Fig. 1). The asymptotic test produces α(x) curves showing the fitted model and bootstrap confidence intervals (right panel of Fig. 1).

## Application

To demonstrate MKado at genome scales, we assembled a dataset of 12,437 human protein-coding genes with both polymorphism and divergence data. Codon-aligned orthologous sequences for human (*Homo sapiens*), chimpanzee (*Pan troglodytes*), and orangutan (*Pongo abelii*) were obtained from OrthoMaM v12 (Allio et al. 2024), which provides one canonical coding-sequence ortholog per gene across mammalian taxa. Human intraspecific polymorphism was drawn from 178 Yoruba (YRI) individuals in the 1,000 Genomes Project NYGC high-coverage dataset (1000 Genomes Project Consortium 2015), yielding 356 phased haplotypes per gene.

For each gene, polymorphism was projected onto the OrthoMaM alignment in five steps: (i) the canonical Ensembl CDS for the gene was reconstructed from the GRCh38 reference and the GTF-derived exon set, providing the mapping from CDS position to genomic coordinate; (ii) the ungapped human row of the OrthoMaM alignment was matched against the Ensembl CDS to obtain the alignment-column to CDS-position correspondence (genes for which this match failed, typically because of curation differences between OrthoMaM and Ensembl, were dropped); (iii) for every CDS position that survived this match, the overlapping YRI variants were extracted from the 1,000 Genomes NYGC VCF, with reference-allele identity confirmed and minus-strand alleles complemented to match the CDS orientation; (iv) per-individual phased haplotypes were assembled from the extracted variants, yielding 356 human haplotype sequences over the Ensembl CDS positions retained by OrthoMaM; and (v) these haplotypes were re-gapped to the OrthoMaM alignment, dropping any human-CDS columns absent from the OrthoMaM alignment and inserting the alignment’s existing gap structure (so insertions, deletions, and non-aligned regions in the multi-species alignment are preserved). Chimpanzee and orangutan sequences were taken directly from the OrthoMaM alignment. Multi-transcript genes are reduced to the single canonical CDS chosen by the OrthoMaM curation; alternative transcripts are not analysed separately. The resulting per-gene multi-species FASTAs are codon-aligned and ready for direct input to MKado.

Using orangutan as the outgroup, genome-wide pooled counts were $D_{n}=94,578$, $D_{s}=188,325$, $P_{n}=44,850$, and $P_{s}=47,838$. The per-gene standard MK test identified numerous genes with significant departures from neutrality, including genes significant after Bonferroni correction (volcano plot, left panel of Fig. 1). As expected, the majority of testable genes showed NI >1, reflecting the well-characterized excess of slightly deleterious nonsynonymous polymorphism in humans (Li et al. 2008). Using chimpanzee as the outgroup yielded fewer fixed differences ($D_{n}=37,120$, $D_{s}=65,228$), roughly 2.5-fold less than orangutan (left panel of Supplementary Fig. S2), and no individual genes survived Bonferroni correction, consistent with the limited power of single-gene tests at shorter divergence times.

The Tarone-Greenland weighted estimate (Stoletzki and Eyre-Walker 2011) across all genes was strongly negative for both outgroups: ${\hat{\alpha }}_{\mathrm{TG}}=-0.853$ (95% CI: −0.888 to −0.812) for orangutan and ${\hat{\alpha }}_{\mathrm{TG}}=-0.651$ (95% CI: −0.682 to −0.607) for chimpanzee, with corresponding ${\text{NI}}_{\mathrm{TG}}$ values of 1.853 and 1.651. These negative values confirm the pervasive excess of weakly deleterious nonsynonymous polymorphism in humans. Excluding singletons shifted both estimates upward (${\hat{\alpha }}_{\mathrm{TG}}=-0.794$ for orangutan; −0.606 for chimpanzee), as expected given the enrichment of slightly deleterious variants at low frequency. Applying a 15% derived allele frequency cutoff (Fay et al. 2001) brought both estimates close to zero (${\hat{\alpha }}_{\mathrm{TG}}=-0.091$ for orangutan; −0.041 for chimpanzee), removing the bulk of low-frequency deleterious variants but still failing to recover a signal of positive selection. Similarly, the aggregated imputed MK test (Murga-Moreno et al. 2022), using a 15% derived allele frequency cutoff, estimated $\hat{\alpha }=-0.097$ for orangutan and $\hat{\alpha }=-0.068$ for chimpanzee, substantially reducing the downward bias but likewise not recovering a positive signal of adaptation.

In contrast, the aggregated asymptotic MK test, which models the frequency dependence of *α* and extrapolates to fixation (Messer and Petrov 2013), successfully recovered a positive signal of adaptive evolution, estimating $\hat{\alpha }=0.163$ (95% CI: 0.075 to 0.253; right panel of Fig. 1) using orangutan as the outgroup, indicating that approximately 16% of amino acid substitutions on the human lineage were driven by positive selection. With chimpanzee as the outgroup the estimate widened to $\hat{\alpha }=0.075$ (95% CI: −1.22 to 0.11; right panel of Supplementary Fig. S2), illustrating the greater uncertainty from the shallower divergence, but also indicating a potential biological signal of less frequent adaptive evolution after the human-Pongo split. These results are consistent with previous analyses of human coding sequences (Messer and Petrov 2013).

The entire analysis—standard MK tests, Tarone-Greenland estimation, asymptotic estimation, imputed estimation, and volcano plot generation for all 12,437 genes—completed in under one minute on a multi-core workstation using MKado’s parallel batch mode.

## Supplementary Material

jkag142_Supplementary_Data

## Data Availability

MKado is freely available at https://github.com/kr-colab/mkado under the MIT license. Full documentation is available at https://mkado.readthedocs.io. MKado is implemented in Python and installable via pip. Supplemental material available at G3 online.
